# Immunization with Tc52 or its amino terminal domain adjuvanted with c-di-AMP induces Th17+Th1 specific immune responses and confers protection against *Trypanosoma cruzi*

**DOI:** 10.1371/journal.pntd.0005300

**Published:** 2017-02-24

**Authors:** Marina N. Matos, Silvia I. Cazorla, Kai Schulze, Thomas Ebensen, Carlos A. Guzmán, Emilio L. Malchiodi

**Affiliations:** 1 Universidad de Buenos Aires, Facultad de Farmacia y Bioquímica, Cátedra de Inmunología and Instituto de Estudios de la Inmunidad Humoral (IDEHU), UBA-CONICET, Buenos Aires, Argentina; 2 Universidad de Buenos Aires, Facultad de Medicina, Departamento de Microbiología, Parasitología e Inmunología and Instituto de Microbiología y Parasitología Médica (IMPaM), UBA-CONICET, Buenos Aires, Argentina; 3 Department of Vaccinology and Applied Microbiology, Helmholtz Centre for Infection Research, Braunschweig, Germany; University of Iowa, UNITED STATES

## Abstract

The development of new adjuvants enables fine modulation of the elicited immune responses. Ideally, the use of one or more adjuvants should result in the induction of a protective immune response against the specific pathogen. We have evaluated the immune response and protection against *Trypanosoma cruzi* infection in mice vaccinated with recombinant Tc52 or its N- and C-terminal domains (NTc52 and CTc52) adjuvanted either with the STING (Stimulator of Interferon Genes) agonist cyclic di-AMP (c-di-AMP), a pegylated derivative of α-galactosylceramide (αGC-PEG), or oligodeoxynucleotides containing unmethylated CpG motifs (ODN-CpG). All groups immunized with the recombinant proteins plus adjuvant: Tc52+c-di-AMP, NTc52+c-di-AMP, CTc52+c-di-AMP, NTc52+c-di-AMP+αGC-PEG, NTc52+CpG, developed significantly higher anti-Tc52 IgG titers than controls. Groups immunized with c-di-AMP and Tc52, NTc52 or CTc52 showed the highest Tc52-specific IgA titers in nasal lavages. All groups immunized with the recombinant proteins plus adjuvant developed a strong specific cellular immune response in splenocytes and lymph node cells with significant differences for groups immunized with c-di-AMP and Tc52, NTc52 or CTc52. These groups also showed high levels of Tc52-specific IL-17 and IFN-γ producing cells, while NTc52+CpG group only showed significant difference with control in IFN-γ producing cells. Groups immunized with c-di-AMP and Tc52, NTc52 or CTc52 developed predominantly a Th17 and Th1immune response, whereas for NTc52+CpG it was a dominant Th1 response. It was previously described that αGC-PEG inhibits Th17 differentiation by activating NKT cells. Thus, in this work we have also included a group immunized with both adjuvants (NTc52+c-di-AMP+αGC-PEG) with the aim to modulate the Th17 response induced by c-di-AMP. This group showed a significant reduction in the number of Tc52-specific IL-17 producing splenocytes, as compared to the group NTc52+c-di-AMP, which has in turn correlated with a reduction in protection against infection. These results suggest that the Th17 immune response developed after immunizing with NTc52+c-di-AMP could have a protective role against *T*. *cruzi* infection. Groups NTc52+c-di-AMP, Tc52+c-di-AMP and NTc52PB, were the ones that showed better protection against infection with lower parasitemia and weight loss, and higher survival.

## Author summary

Chagas disease is a parasitic disease caused by a protozoan parasite (*Trypanosoma cruzi*) which has a complex life cycle including insect vector and mammalians. In Latin America, 7–10 million people are infected, 100 million people are at risk of infection, and about 56,000 new infection cases and 12,000 deaths are registered annually. Migration spread the geographic distribution of the disease to North America and Europe. The infection in humans has an initial acute stage followed by a chronic stage where up to 30% of patients develop cardiac alterations and 10% develop digestive, neurological or mixed alterations. The acute infection is hardly detected and there is not drug to treat the chronic infection. Thus, there is an urgent need for prophylactic and therapeutic vaccines development. Several attempts to find a vaccine antigen has been made and the protein Tc52 is a good candidate. In a vaccine composition, as important as the antigen is the adjuvants, which are substances able to increase, improve or modified the immune response. This research provides information about the immune response and protection against *Trypanosoma cruzi* infection elicited by Tc52 or portions of this molecule using different adjuvants.

## Introduction

Recent estimations indicate that about 6–7 million people are infected worldwide with *Trypanosoma cruzi* [1], a protozoan parasite that is the etiological agent of Chagas disease [2]. Vectorial transmission takes place in endemic countries where the vector, a triatomine insect, is present. In Latin America, one hundred million people are at risk of infection, and about 56,000 new infection cases and 12,000 deaths are registered annually [3]. Geographic distribution of Chagas disease spread in the last decades due to migration, with more than 300,000 people being infected in the United States, as indicated by CDC estimations [4]. *T*. *cruzi* new infections in North America, Europe and Asia are mainly the consequence of transfusion of contaminated blood, congenital transmission and organ transplantation.

The infection has an initial acute stage followed by a chronic stage where up to 30% of patients develop cardiac alterations and 10% develop digestive, neurological or mixed alterations [1, 5]. Currently, only two drugs are used for treatment: Nifurtimox and Benznidazole. Both are effective in the acute stage of infection, lose effectiveness in the advanced phase, and have important side effects associated to the treatment [5, 6]. Therefore, efforts are focused not only in transmission control and the search for more efficient and less toxic drugs, but also in the development of prophylactic and therapeutic vaccines.

In the last years, vaccine development was focused on some antigens that proved to be promising candidates employing different adjuvants, immunization protocols and strategies, including recombinant proteins, DNA vaccines and viral vectors (reviewed in [7, 8, 9]). Tc52 is a *T*. *cruzi* protein with glutathione transferase activity [10] which has two domains with similar size: the N-terminal (NTc52) and the C-terminal (CTc52) domains [11]. Many immunomodulatory properties were described for Tc52 that make it an interesting target for vaccine development. It binds to macrophages and dendritic cells and, in the presence of IFN-γ, it induces iNOS expression and NO synthesis by macrophages [12]. Also, Tc52 inhibits the proliferation of splenocytes induced by mitogen, an activity for which probably the C-terminal domain is responsible [13, 14]. Tc52 is essential for the parasite, since the double knock-out is lethal [15]. Thus, Tc52 seems to be a promising vaccine candidate against *T*. *cruzi* infection. Therefore, vaccine prototypes were assayed using this antigen [16, 17].

We have also tested Tc52 as a vaccine candidate using different protocols. We demonstrated that NTc52 gives better protection than CTc52 and similar to full-length Tc52 when assayed as a DNA-delivery system carried by attenuated *Salmonella* [18]. We then evaluate the role of Tc52 DNA in a multi-component vaccine prototype with promising results [19]. We also developed a DNA-prime/protein-boost vaccine strategy focusing on NTc52 [20]. In this work, we look for a safe vaccine with higher acceptance. To this end, instead of a DNA-based vaccine using an attenuated bacterium as carrier, we use purified protein Tc52 produced in *Pichia pastoris*, an organism with GRAS (Generally Recognized As Safe) status conferred by the USA FDA [21, 22].

Since infection by *T*. *cruzi* could take place by vector-mediated breaks in skin and mucosa, by transfusions and organ transplants, by congenital infection and by oral route through the gastrointestinal tract [23], we analyzed vaccine candidates that induce both systemic and mucosal immunity. The novel mucosal adjuvant bis-(3’,5’)-cyclic dimeric adenosine monophosphate (c-di-AMP) is a bacterial second messenger, involved in sensing DNA integrity, control of cell wall and potassium homeostasis [24]. This compound promotes expression of type I IFN and TNF through the activation of a STING/TBK1 axis. The co-administration of an antigen and c-di-AMP by intranasal route in BALB/c mice induced both systemic and mucosal antibody responses, and a balanced Th1/Th2/Th17 cellular specific immunity pattern [25]. Another mucosal adjuvant, a pegylated derivate of the α-galactosyl-ceramide (αGC-PEG), induces a dominant Th2 immune response [26], and more interestingly, it inhibits Th17 cell differentiation by activating NKT cells [27]. The role of IL-17 and Th17 cells in *T*. *cruzi* infection are still not well characterized. Though, reports suggested that IL-17 could have a protective role in infection and tissue damage control [28–32]. In this work we use c-di-AMP as an adjuvant for intranasal immunization with the recombinant proteins Tc52, NTc52 and CTc52. Also, we test the co-administration of NTc52 together with c-di-AMP and αGC-PEG to evaluate the ability of αGC-PEG to block Th17 response and its effect in the conferred protection against *T*. *cruzi* infection.

## Materials and methods

### Parasite

*T*. *cruzi* epimastigotes (RA strain) were grown in LIT medium as previously described [18]. The *T*. *cruzi* bloodstream trypomastigotes (RA strain) and the recombinant Tulahuen strain expressing β-galactosidase (Tul-β-Gal) [33] were isolated from the blood of infected mice.

### Tc52, NTc52 and CTc52 cloning and expression in *Pichia pastoris*

Full-length Tc52 and its N-terminal domain (NTc52) were cloned and expressed in the yeast *P*. *pastoris* using the pPICzα-A expression vector as previously described [20]. Tc52 C-terminal domain (CTc52), corresponding to the Tc52 amino acid residues 224 to 245 [11, 18], was similarly cloned and expressed. The DNA sequence encoding for CTc52 was amplified from *T cruzi* RA strain epimastigote genomic DNA and cloned in the pPICzα-A vector. For that purpose the following oligonucleotides were used: the forward primer 5’CGACTGGAATTCGCTCCTGGCTATGTACTTTTTGTT3’ containing an *EcoR*I restriction site (underlined), and the reverse primer 5’ACTAGCGCGGCCGCTCAGTGATGGTGATGGTGATGCAATGACCATGTGACGTGC3’, with a *Not*I restriction site and a sequence encoding a His_6_ tag (both underlined). Cloning, clone selection, and protein expression and purification were performed as described for NTc52 and Tc52 [20].

### Cloning and expression of NTc52 using a eukaryotic expression system

Cloning and expression of NTc52 in pcDNA3.1 plasmid, and transformation of attenuated *Salmonella enterica* serovar Typhimurium *aroA* SL7207, was performed as previously described [18].

### Immunizations and challenge

For the immune response analysis, nine groups (6 animals/group) of inbred female 6- to 8-week-old C3H/HeN (H-2K haplotype) mice were immunized 3 times every 10 days by intranasal (i.n.) route. Group I (GI): Control (received PBS alone); GII: Tc52, GIII: NTc52, GIV: CTc52, GV: Tc52+c-di-AMP, GVI: NTc52+c-di-AMP, GVII: CTc52+c-di-AMP, GVIII: NTc52+c-di-AMP+αGC-PEG, GIX: NTc52+ODN-CpG. Mice were immunized with the same number of molecules (0.42 nmol) of each recombinant protein according to the predicted molecular weight: Tc52 (52.2 kDa) 21.9 μg, NTc52 (26.9 kDa) 11.3 μg, CTc52 (25.6 kDa) 10.7 μg. The amounts of adjuvants used per mice were: c-di-AMP 5 μg, αGC-PEG 15 μg, and ODN-CpG 1826 20 μg. These amounts were selected according to previous results [20, 25, 28, 34, 35].

To analyze the protection conferred against *T*. *cruzi* challenge, seven groups of mice (5 animals/group) were immunized 3 times every 10 days as follows: GI: control with c-di-AMP alone, GII: control with αGC-PEG alone, GIII: Tc52+c-di-AMP, GIV: NTc52+c-di-AMP, GV: CTc52+c-di-AMP, GVI: NTc52+c-di-AMP+αGC-PEG, GVII: NTc52+αGC-PEG, and GVIII: NTc52PB. We included the group GVIII as positive control, since it was our previous best prime-boost protocol using the antigen Tc52 [18, 20]. Briefly, GVIII mice were immunized with 2 oral doses of 10^9^ CFU of *Salmonella* SL7207 delivering the construction pcDNA3.1-NTc52 (SNTc52), followed by 2 doses of NTc52+ODN-CpG 1826 by intradermal route. To ensure that the last immunization was at the same time for all groups, the immunization protocol for GVIII started 10 days before the other groups. Twenty days after the last immunization, mice were infected by intraperitoneal route with 10^3^ *T*. *cruzi* bloodstream trypomastigotes of the highly virulent RA strain.

Experiments with animals were approved by the Review Board of Ethics of the Faculty of Pharmacy and Biochemistry (UBA, Argentina) and in agreement with the local government of Lower Saxony, Germany; and conducted in accordance with the guidelines established by the National Research Council [36].

### Antibody determination

Serum samples, nasal lavages and saliva samples were collected 20 days after the last immunization for the measurement of antigen-specific IgG and IgA, respectively. Measurement of antigen-specific IgG and IgA was carried out by Enzyme Linked Immune Assays (ELISA) as described previously [18]. Plates were coated with recombinant Tc52 (rTc52, 2 μg/ml). For the measurement of total IgA in nasal lavages, plates were coated with anti-IgA polyclonal antibody (2 μg/ml). IgA specific titers were normalized to 10 μg of total IgA/well.

### Proliferation assays

Proliferation of spleen and cervical lymph nodes cells after stimulation with antigen rTc52 was evaluated by ^3^H-thymidine incorporation as previously described [18]. The results were expressed as proliferation index (PI), defined as the cpm (counts per minute) in the presence of the antigen/cpm in the absence of the antigen.

### Enzyme-Linked Inmuno Sorbent SPOT(ELISPOT)

The number of IFN-γ, IL-4 and IL-17 producing spleen cells in response to antigenic stimulus was determined by ELISPOT. Ninety-six well plates were sensitized with capture antibodies (anti-IFN-γ, anti-IL-4, or anti-IL-17) for 16 h, at 4°C, and then blocked (with RPMI medium supplemented with 10% fetal bovine serum, FBS). Splenocytes (2 x 10^5^ or 4 x 10^5^ cells/well) were added in presence or absence of Tc52 (0.5 μg/well), and incubated at 37°C, 5% CO_2_, for 24 h for IFN-γ and 48 h for IL-4 and IL-17. Plates were washed and then incubated (2h, 25°C) with biotinylated detection antibody and then with Avidin-HRP. As peroxidase substrate, 3-amino-9-ethil-carbazole (AEC) was used. Plates were scanned and spots were quantified in an ImmunoSpot CTL. Results were expressed as number of spots for 10^6^ cells.

### Blood parasite levels, survival and weight monitoring

Twenty days after the last immunization, mice were infected by intraperitoneal route with 1000 *T*. *cruzi* bloodstream trypomastigotes of the highly virulent RA strain. Weight and parasitemia were measured every 2 or 3 days after the challenge as reported [18].The change in weight was expressed as a percentage (W%) and calculated as follows: W% = (Wi—Wo) x 100%/Wo, where Wo is the weight of each mouse immediately before infection, and Wi is its weight at day i post-infection. Survival was monitored daily.

### Statistical analysis

Statistical analyses were carried out with Prism software version 5.0 (GraphPad, San Diego, CA), and R [37]; using a nonparametric Kruskal-Wallis test and Dunn’s posttest. The survival curves were analyzed with a log rank Mantel-Cox test. All the comparisons were done in reference to control groups (PBS, c-di-AMP or αGC-PEG), except when indicated. P values of less than 0.05 were considered significant.

## Results

### All tested adjuvants enhance systemic antigen-specific antibody response in vaccinated mice

**Fig 1:**
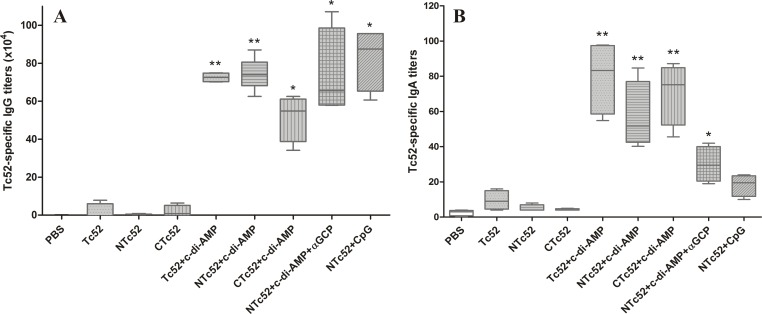
Antibody response against Tc52 in immunized mice. Sera and nasal lavage of each mouse were taken 20 days after the last immunization. **A.** Tc52-specific IgG ELISA titers in sera against immobilized full-length Tc52 (2 μg/ml). **B.** Tc52-specific IgA in nasal lavage samples determined by ELISA. Total IgA in each sample was measured by capture ELISA and the titer for each sample was then normalized to 10 μg of total IgA/well. Results are showed in box plots, line correspond to median, boxes to 25^th^ and 75^th^ percentiles, and whiskers to minimum and maximum values. Results are representative of three independent experiments. *P < 0.05; **P < 0.01.

The antigen specific antibody responses developed after immunization were analyzed by ELISA in sera of all mice immunized. Plates were sensitized with full-length Tc52 instead of the immunizing antigen (Tc52, NTc52 or CTc52), to evaluate the elicited antibodies that recognize their cognate epitope in the molecule exposed by the parasite. Mice immunized with recombinant proteins (Tc52, NTc52 and CTc52) plus c-di-AMP, and those immunized with NTc52 plus c-di-AMP+αGC-PEG or ODN-CpG, developed high titers of Tc52-specific IgG antibodies with significant differences compared to the control group. Mice immunized just with the proteins in the absence of any adjuvant did not show antibodies titers different than mice receiving PBS (**Fig 1A**).

### The adjuvant c-di-AMP induced high titers of antigen specific IgA in nasal mucosa

Our main objective by giving the immunogen by intranasal route was to generate mucosal immunity including antibodies able to block the entrance of parasites by this route. Groups immunized with Tc52, NTc52 or CTc52 in the presence of c-di-AMP showed titers of Tc52-specific IgA in nasal lavages which were significantly higher than in controls (**Fig 1B**). Even when both c-di-AMP and αGC-PEG are potent mucosal adjuvants [25, 26, 34], group VIII (NTc52+c-di-AMP+αGC-PEG) developed lower specific IgA titers than mice in the group VI (NTc52+c-di-AMP). Nevertheless, no significant difference between these groups was observed. Although the ability of ODN-CpG as mucosal adjuvant was widely demonstrated [38–40], we found that group IX (NTc52+CpG) developed anti-Tc52 IgA titers higher than the control (median 19.5 and 3.0, respectively), but the difference was not significant. This result corroborates our previous report that ODN-CpG is not able to elicit mucosal IgA against Tc52 [20].

### Immunizations with recombinant proteins plus the adjuvants induce strong specific cellular immune response

**Fig 2:**
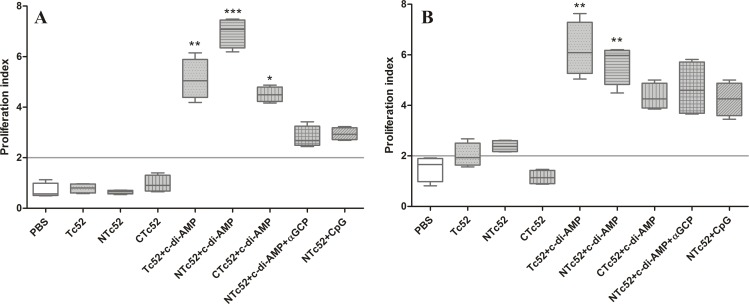
Cellular immune response against Tc52 in vaccinated mice. Lymph nodes and spleen were taken from each mouse 20 days after the last immunization. Proliferative responses are expressed as proliferation index (PI): a ratio of [^3^H]-thymidine incorporation (cpm) between cells stimulated with Tc52 and non-stimulated in **A.** lymph nodes cells, and **B**. spleen cells. Results are shown in box plots, line corresponds to median, boxes to 25^th^ and 75^th^ percentiles, and whiskers to minimum and maximum values. Results are representative of three independent experiments. *P < 0.05; **P < 0.01; ***P < 0.001.

The Tc52-specific cellular immune response was studied *ex vivo* in splenocytes and lymph nodes cells by proliferation assays (**Fig 2**). Mice from groups V to IX developed proliferative responses upon *in vitro* Tc52 reestimulation in splenocytes and in lymph node cells. For lymph node cells (**Fig 2A**) differences to the control were significant for GV (p < 0.01), GVI (p < 0.001) and GVII (p < 0.01), whereas only GV (Tc52+c-di-AMP) and GVI (NTc52+c-di-AMP) spleen cells showed significant differences with respect to the control group (p < 0.01) (**Fig 2B**).

### Immunization with c-di-AMP induces a Th17+Th1 cellular immunity pattern

As expected, c-di-AMP induced an immune response activating Th1, Th2 and Th17 cells [25]. On the other hand, αGC-PEG inhibits Th17 cell differentiation by activating NKT cells [26, 27]. Thus, we co-administered the adjuvants c-di-AMP and αGC-PEG in order to selectively block with the second adjuvant the Th17 response stimulated by the first one.

**Fig 3:**
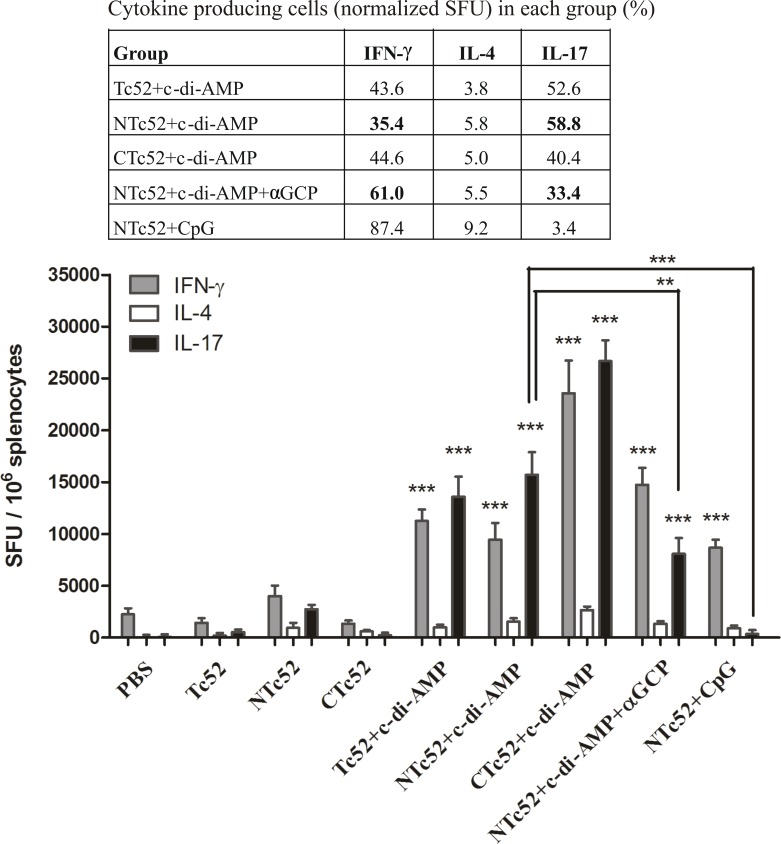
Cytokine profile developed after immunizations in vaccinated mice. Spleen cells were taken from each mouse 20 days after the last immunization. After stimulation with Tc52, IFN-γ, IL-4 and IL-17 producing cells were measured by ELISPOT. Results are expressed as spot forming units (SFU) normalized to 10^6^ spleen cells, showing the mean and standard deviation of each group. In the table, the mean of normalized SFU of each cytokine is expressed as percentage of the sum of the three cytokines measures in the group to estimate the relative secretion of each cytokine, no the cells producing them. The change in cytokine profile in immunizations with c-di-AMP, with or without αCGP, is highlighted in bold. Results are representative of two independent experiments. **P < 0.01; ***P < 0.001.

With the aim to evaluate the type of immune response developed by the different immunization protocols, we focused on three cytokines: IL-17, IFN-γ and IL-4. To this end, the number of cytokine-producing cells in the presence of rTc52 was determined by ELISPOT. We found that recombinant proteins adjuvanted with either ODN-CpG or c-di-AMP elicited cells able to strongly secrete IFN-γ after stimulation. These were significantly more abundant (p<0.001) than in animals receiving the recombinant proteins without adjuvant (**Fig 3**). When IL-17 was analyzed, every group adjuvanted with c-di-AMP elicited cells that strongly secreted IL-17 upon restimulation with rTc52. In contrast, the group adjuvanted with ODN-CpG was not able to induce IL-17-secreting cells.

When the result of each cytokine was analyzed as percentage of the sum of the three cytokines in each group, we found that the profile of cytokine-producing cells was similar for groups immunized with c-di-AMP containing vaccines, with values between 35–45% for IFN-γ and 40–59% for IL-17. In contrast, groups immunized with ODN-CpG containing vaccines induced predominantly IFN-γ with ~88% of the total and practically no secretion of IL-17. These results highlight the significant influence of c-di-AMP on the stimulation of a mixed Th17- and Th1-oriented immune response.

Another important result was observed in mice immunized with NTc52+c-di-AMP+αGC-PEG, where we confirm that IL-17 secretion was partially blocked by the use of αGC-PEG as co-adjuvant (**Fig 3B**), when comparing to mice immunized with NTc52+c-diAMP (p < 0.01).

### The immunization with recombinant proteins and adjuvants protects against *T*. *cruzi* lethal challenge

The groups that were included in the immunoprotection study differ little from those used to assess the immune response. Since immunization with NTc52 and CTc52 recombinant proteins without adjuvant did not induce a significant immune response, these groups were excluded from the evaluation of protection against parasite infection. However, two additional groups were included: (i) the NTc52+αGC-PEG group, to evaluate the protection generated by the NTc52 protein plus the αGC-PEG adjuvant alone, which induces a Th2-dominated immune response [26], and (ii) a positive control group primed orally with attenuated *Salmonella* delivering the construction pcDNA3.1-NTc52 (SNTc52), followed by a boost of 2 doses of NTc52+ODN-CpG 1826 by intradermal route. This group (NTc52PB) was included as gold standard because it provided the best protection in previous experiments [20].

**Fig 4:**
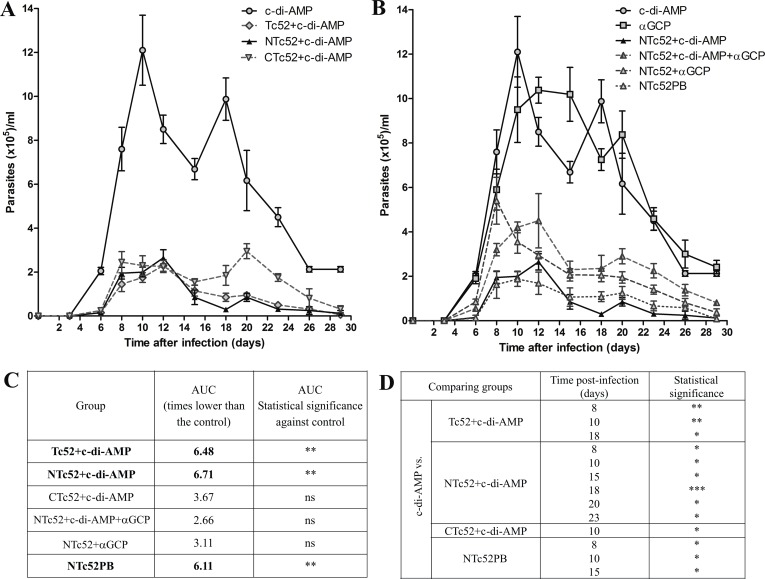
Parasitemia after lethal *T*. *cruzi* challenge. Twenty days after the last immunization, mice were lethally challenged with the 1000 RA strain bloodstream trypomastigotes. Levels of parasites in blood were measured in each mouse every 2 or 3 days. **A.** Mice were immunized by intranasal route with Tc52+c-di-AMP, NTc52+c-di-AMP, or CTc52+c-di-AMP, and the control group received c-di-AMP alone. **B.** Groups immunized with NTc52 and different adjuvants: NTc52+c-di-AMP, NTc52+c-di-AMP+αGCP, NTc52+αGCP, a prime-boost strategy: NTc52PB (previously described in Matos 2016), and both control groups (c-di-AMP and αGCP). **C.** Area under the curve (AUC) of parasitemia of all immunized groups related to AUC of control group (times lower than control). c-di-AMP was taken as control group, except for group NTc52+αGCP. For group NTc52+c-di-AMP+αGCP, the comparison was done with both controls (c-di-AMP and αGCP), with similar results. **D.** Statistical analysis at each point of parasitemia curves. Statistical significance between control group (c-di-AMP) and other groups is indicated. Results are representative of three independent experiments. *P < 0.05; **P < 0.01; ***P < 0.001; ns: non-significant.

All immunized mice developed less parasitemia than control groups (**Fig 4**). **Fig 4A** shows the strong protection induced by Tc52 and its domains when c-di-AMP is used as adjuvant. When the area under the curve (AUC) is analyzed, the full-length antigen and the N-terminal domain showed lower AUC with significant difference against control (c-di-AMP), p > 0.01, with non parametric test (**Fig 4C**). AUC from group CTc52+c-di-AMP did not show significant difference against control. **Fig 4B** shows the comparison of the adjuvants and protocols when the antigen used is NTc52, which strongly reduces parasitemia. The reduction was especially noticeable for groups NTc52+c-di-AMP (GIV), Tc52+c-di-AMP (GIII) and NTc52PB (GVIII), which showed AUCs 6.71, 6.48 and 6.11 times lower than the control group, respectively.

The protection in terms of parasitemia was also analyzed at each point with nonparametric statistical tests (**Fig 4D**). All immunized groups except NTc52+αGC-PEG and NTc52+c-di-AMP+αGC-PEG present significant differences compared to the control in at least one point. Groups that show better protection were the same as when AUCs were analyzed: GIII, GIV and GVIII. Taken both analyses together, the groups that showed better protection were: NTc52+c-di-AMP, Tc52+c-di-AMP and NTc52PB. Also, it is notable that αGC-PEG is not a good adjuvant for developing vaccines against *T*. *cruzi* infection using NTc52 as antigen. Group NTc52+αGC-PEG showed an AUC 3.11 times lower than control, and no significant differences, whereas the other immunization protocols with NTc52 gave better protection. Importantly, immunization with NTc52 in the presence of both adjuvants (αGC-PEG and c-di-AMP) induces less protection than immunizing with only c-di-AMP, suggesting the potential relevance of IL-17 in the control of *T*. *cruzi* infection when c-di-AMP is used.

**Fig 5:**
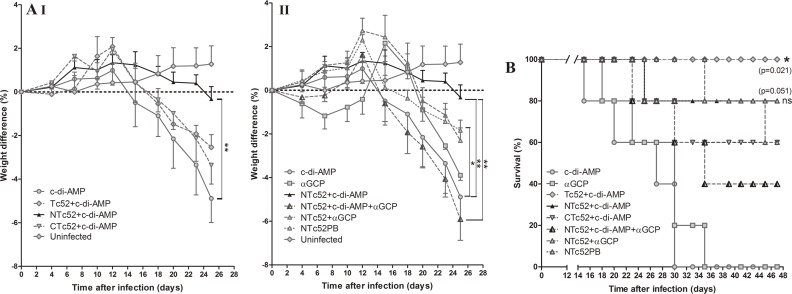
Weight change and survival after infection. **A.** Mouse weight was determined every 2 or 3 days after a lethal *T*. *cruzi* challenge, and the weight difference was recorded for each group. Mice weight in uninfected mice is also shown during the time. Statistical significance at 25 dpi is indicated. **I.** Groups immunized with recombinant proteins and c-di-AMP as adjuvant: Tc52+c-di-AMP, NTc52+c-di-AMP, CTc52+c-di-AMP and control (c-di-AMP). **II.** Groups immunized with NTc52 and different adjuvants: NTc52+c-di-AMP, NTc52+c-di-AMP+αGCP, NTc52+αGCP, a prime-boost strategy: NTc52PB (previously described in Matos 2016) and control groups (c-di-AMP and αGCP). **B.** Survival was monitored daily. Results are representative of three independent experiments. *P <0.05; **P < 0.01.

To analyze the protection conferred by vaccination, we also follow up the animal body weight every 2–3 days after infection (**Fig 5A**). All immunized groups showed less weight loss than the control group. **Fig 5AI**shows that c-di-AMP by itself is unable to reduce the weight loss induced by infection. This adjuvant plus Tc52 or CTc52 was able to reduce the weight loss only in a small proportion of the animals. However, the reduction of weight loss was really significant (p < 0.01) when c-di-AMP was used in combination with NTc52. **Fig 5AII**shows the comparison of the adjuvants and protocols when the antigen used is NTc52. At 25 dpi, the difference was significant only for the groups NTc52+c-di-AMP and NTc52PB (p < 0.05);the NTc52+c-di-AMP group being the one that showed less body weight loss, behaving closely to uninfected mice. Moreover, at 25 dpi, this group also showed significant difference in body weight change with respect to the group vaccinated with NTc52+c-di-AMP+αGC-PEG (p < 0.05), in which a strong weight loss was observed, even higher than in controls immunized with just adjuvant.

Survival was monitored daily post infection. All mice from both control groups (c-di-AMP and αGC-PEG) died by 35 dpi, whereas the survival of the other groups was 100% for Tc52+c-di-AMP, 80% for NTc52+c-di-AMP and NTc52PB, 60% for CTc52+c-di-AMP, and 40% for NTc52+c-di-AMP+αGC-PEG (**Fig 5B**). Group NTc52+αGC-PEG showed 80% survival until 45 dpi, after which it fell to 60%. Survival remains unchanged until 100 dpi, when mice were sacrificed. Survival was significantly different between the control group and the Tc52+c-di-AMP group (p<0.05), whereas no significant differences were seen between the control group and the NTc52+c-di-AMP or NTc52PB groups (p = 0.051).

## Discussion

In this research project, we focused on the development of an intranasal vaccine, seeking to stimulate both systemic and mucosae pathogen-specific protective immunity. Previously, we demonstrated that the N-terminal domain of Tc52 (NTc52) is a promising vaccine candidate [18, 20]. Here, we have focused on this subunit administered with c-di-AMP as adjuvant, but we also evaluated the protective role of the adjuvant with full-length Tc52 and CTc52 as recombinant proteins.

Twenty days after the last immunization, all groups that received a recombinant protein plus any of the adjuvants (c-di-AMP, c-di-AMP+αGC-PEG or ODN-CpG) developed high anti-Tc52 IgG titers, which were significantly different than in controls, as expected according to previous reports [25, 26, 34, 41]. Nevertheless, the results were different when we analyzed the mucosal immunity elicited. Only mice immunized with Tc52, NTc52 or CTc52 co-administered with c-di-AMP developed high Tc52-specific IgA titers in nasal lavages, showing the superior ability of c-di-AMP to induce mucosal immunity [25, 34]. The group immunized with NTc52+c-di-AMP+αGC-PEG developed high IgA specific titers, significantly different than the controls. These titers were lower than in the NTc52+c-di-AMP group, but without statistical significance. We find that the use of c-di-AMP and αGC-PEG together did not improve mucosal immunity. Group NTc52+CpG developed anti-Tc52 IgA titers higher than the controls, but without significant differences. That contrasts to other reports that used ODN-CpG as an adjuvant in mucosal vaccines [39, 40], but correlates to our previous results observed in mice immunized with NTc52+CpG [20].

We have also analyzed cellular immune responses *ex vivo* in splenocytes and lymph node cells. Cells from all groups immunized with Tc52, NTc52 or CTc52, adjuvanted with c-di-AMP, αGC-PEG+c-di-AMP or ODN-CpG, showed a proliferation index over 2. However, only groups immunized with c-di-AMP (Tc52+c-di-AMP, NTc52+c-di-AMP, CTc52+c-di-AMP) presented significant differences with respect to controls. The ability of c-di-AMP as mucosal adjuvant to induce specific cellular immunity was already described [25, 34]. Nevertheless, this is the first work in which immunization with a protein antigen plus c-di-AMP or ODN-CpG (and also c-di-AMP+αGC-PEG) are tested together. In our protocol, using the same antigen (NTc52), c-di-AMP induced stronger cellular and humoral immune responses than ODN-CpG, as demonstrated by antigen-specific IgG and IgA titers, as well as proliferation assays.

The profile of the cellular immune response was analyzed in splenocytes by ELISPOT, studying IFN-γ, IL-4 and IL-17 producing cells. Immunizations with c-di-AMP showed a cytokine profile related to a mixed Th17+Th1 immune response for all the antigens studied. In contrast, in mice receiving ODN-CpG the immune response elicited was predominantly Th1. The co-administration of c-di-AMP and αGC-PEG with NTc52 induced a cytokine-producing cells profile of IFN-γ > IL-17 > IL-4. The number of IL-17 spot forming units (SFU) was significantly different for groups NTc52+c-di-AMP and NTc52+c-di-AMP+αGC-PEG, showing the ability of αGC-PEG to inhibit, at least partially, the Th17 immune response induced by c-di-AMP. Ebensen *et al*. [26] have previously described in BALB/c mice (H-2d) immunized by i.n. route with β-galactosidase + αGC-PEG, a cellular specific profile of IL-4 > IFN-γ (IL-17 was not assayed). These differences might be explained not only by the use of a different antigen, but also by the differences in the mouse strain. It is well known that BALB/c mice develop predominantly a Th2 profile, whereas C3H/HeN (H-2k) mice present a more balanced Th1/Th2 immune response.

Protection against infection was evaluated challenging the immunized mice with a lethal dose of the highly virulent *T*. *cruzi* RA strain. The challenge was conducted intraperitoneally as a heterologous route for a more rigorous test of vaccine efficacy. Immunized mice showed protection as compared with controls, in terms of parasitemia, weight loss and survival. However, the groups Tc52+c-di-AMP, NTc52+c-di-AMP and NTc52PB were the ones that showed higher protection with significant differences with respect to the control groups in almost all parameters tested. In this case, we were more strict and analyzed if the protection elicited by immunization with c-di-AMP was able to improve the previous reported by us performance in a heterologous DNA-prime/protein-boost protocol, NTc52PB [20].We find that NTc52+c-di-AMP is the group that stands better in all parameters, particularly in reduction of parasitemia levels and reduction of weight loss, showing not only significant differences alongside controls (p<0.01), but also similar weight changes compared to uninfected mice, with almost no reduction in body weight at 25 dpi. For comparisons it must be beared in mind that the NTc52PB protocol not only includes 4 doses (one more than the other groups), but also involves the immunization with DNA and protein, and by different routes (i.e. a more cumbersome protocol associated with considerable production constraints and difficult implementation logistics).

As was expected, immunization with NTc52+αGC-PEG induced partial protection against infection since αGC-PEG, like α-GC, activates NKT cells and leads to a Th2 biased immune response [26, 27, 42], whereas protection against *T*. *cruzi* infection is provided by a Th1 immune response [43, 44].

We found that the Tc52 N-terminal domain represents a more protective antigen, giving the same or even more protection than the full-length Tc52 and clearly more than the C-terminal domain. The pattern of cytokine-producing cells was similar for those 3 groups (Tc52+c-di-AMP, NTc52+c-di-AMP and CTc52+c-di-AMP): predominantly IL-17 and IFN-γ. This shows the notable influence of this adjuvant in the type of immune response developed. IL-17 is a pro-inflammatory cytokine which has a protective role against infection by many pathogens, inducing neutrophil recruitment as well as secretion of pro-inflammatory soluble factors. The role of IL-17 and Th17 cells in *T*. *cruzi* infection is still not well characterized. Nevertheless, reports suggested that IL-17, secreted by Th17 or other cells, could have a protective role in infection and tissue damage control. Da Matta Guedes et al. [28] have also described the protective role of IL-17 modulating Th1 differentiation in the heart, and thus, controlling myocarditis. In accordance with that, recent reports demonstrate a correlation between IL-17 levels and cardiac function in patients with Chagas disease [32]. Furthermore, IL-17^-/-^ mice infected with *T*. *cruzi* showed lower survival and higher blood levels of parasite and aspartate aminotransferase (AST, a marker of muscle damage), than wild type mice [29]. A recent study with knock out mice for the cytokine receptor IL-17 RA demonstrated the important role of this receptor in protection against *T*. *cruzi* infection, recruiting IL-10-producer neutrophils that could modulate the IFN-γ-dependent inflammatory response, and thus, tissue damage. In *T*. *cruzi* infection, IL-17 could be produced by many cell types, including NKT, Tγδ, CD4^+^ Th17 and CD8^+^cells [30].More recently, B cells were identified as the principal source of IL-17 in *T*. *cruzi* infection [31].

In this work we have studied the role of different immune response patterns, developed by different immunization strategies, in *T*. *cruzi* infection focusing on Th17 stimulation. We perform immunizations by intranasal route with the aim of inducing both systemic and mucosal immunity. Also, this immunization route induces *per se* a Th17 immune response [27].This is the first work in which the immunization with an antigen and c-di-AMP or c-di-AMP + αGC-PEG is used in the development of a vaccine against a parasite infection, evaluating both the immune response and protection developed. It was demonstrated that αGC-PEG changes the cytokine profile induced by c-di-AMP, partially reducing the number of antigen-specific IL-17 producing cells in spleen. This change in the elicited immune response correlates with a sharp reduction in protection against infection, confirming the role of Th17 response in protection against *T*. *cruzi* infection. Closely related to our results, in a recent work the importance of both Th1 and Th17 immune responses for vaccine-induced immunity against *Mycobacterium tuberculosis* (an intracellular pathogen) was suggested [45].

This work emphasizes the importance of the Th17 immune response developed in the groups adjuvanted with c-di-AMP. Previously [20], and also in this work, we have demonstrate that NTc52 in a DNA-prime/protein-boost vaccine strategy confers clear protection against *T*. *cruzi* infection. In the previous work, we focused on the Th1-Th2 immune response; the IL-17 specific response was not analyzed. In this work we have focused on c-di-AMP adjuvanting Tc52, NTc52 and CTc52, and also the modulation of the immune response by αGC-PEG. As we mentioned before, we have included the NTc52PB group as a gold standard in challenge assays, because in previous reports it was our best prototype vaccine based on Tc52 as antigen [20].

As concluding remarks, our studies highlight the potential of intranasal immunization with NTc52+c-di-AMP, which induced strong cellular and antibody (both mucosae and systemic) responses with a Th17+Th1 profile, as a prophylactic strategy against Chagas. This immunization protocol conferred indeed clear protection against lethal infection, as demonstrated by the parasitemia, survival and weight loss parameters.
